# 

**Published:** 1896-10-24

**Authors:** 


"The standard of highest purity at present attainable."—Lancet.

CADBURY'S COCOA

NO ALKALIES USED.

### THE HOSPITAL

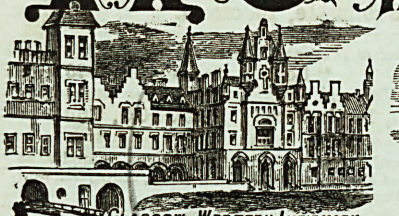

Glasgow Western Infirmary

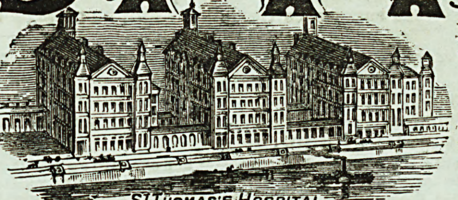

St Thomas's Hospital

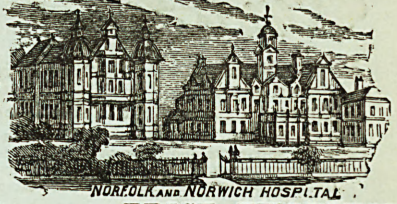

Norfolk and Norwich Hospital

No. 526. Vol. XXI.

Saturday, Oct. 24th, 1896.

WITH EXTRA NURSING SUPPLEMENT.

[Registered at the General Post Office as a Newspaper for transmission in the United Kingdom and Abroad.]

PRICE TWOPENCE.

Per Annum, 10s. 6d., post free.

Monthly Parts, 6d.; post free, 8d.

Ditto per Annum, 8s. 6d., post free.

